# A Scoping Review of Graphic Medicine Interventions to Promote Changes in Health Behavior, Health Service Engagement, and Health Outcomes

**DOI:** 10.3390/ijerph22050657

**Published:** 2025-04-22

**Authors:** Sarah Febres-Cordero, Athena D. F. Sherman, Biyeshi Kumsa, Meredith Klepper, Fawas Shanun, Sophie Grant, Brenice Duroseau, Sharon L. Leslie, Pranav Gupta, Abigail Béliveau, Patti Landerfelt, Sydney Cohen, Carissa Lawrence, Whitney Linsenmeyer, Molly Szczech, Monique S. Balthazar, Don Operario

**Affiliations:** 1 Nell Hodgson Woodruff School of Nursing, Emory University, Atlanta, GA 30322, USA; adfsherman@emory.edu (A.D.F.S.); sophie.grant@emory.edu (S.G.); patti.e.landerfelt@emory.edu (P.L.); sydney.cohen3@emory.edu (S.C.); molly.szczech@emory.edu (M.S.); 2 Rollins School of Public Health, Emory University, Atlanta, GA 30322, USA; biyeshikumsa9@gmail.com (B.K.); fawaz.shanun@emory.edu (F.S.); don.operario@emory.edu (D.O.); 3 School of Nursing, Johns Hopkins University, Baltimore, MD 21218, USA; mkleppe1@jhmi.edu (M.K.); bdurose1@jhmi.edu (B.D.); clawre15@jhmi.edu (C.L.); 4 Woodruff Health Sciences Center Library, Emory University, Atlanta, GA 30322, USA; sharon.leslie@emory.edu; 5 School of Medicine, Emory University, Atlanta, GA 30322, USA; pranav.gupta@emory.edu; 6 School of Nursing, University of North Carolina at Chapel Hill, Chapel Hill, NC 27599, USA; ajulier@ad.unc.edu; 7 Nutrition and Dietetics, Saint. Louis University, Saint. Louis, MO 63103, USA; whitney.linsenmeyer@health.slu.edu; 8 Ross and Carol Nese College of Nursing, Pennsylvania State University, University Park, PA 16802, USA; msb6243@psu.edu

**Keywords:** graphic medicine, art, comics, intervention design, intervention mediums, health literacy, equity

## Abstract

Low health literacy is a known contributing factor to poorer patient outcomes. Health information is often presented through materials written at high reading levels and thus may be an ineffective education tool for patients of diverse socioeconomic backgrounds, age ranges, and education levels. Graphic medicine (i.e., healthcare concepts presented through illustrations, such as comics or cartoons) may be a more equitable and efficacious format for many patients. The purpose of this review was to describe the efficacy and use of graphic medicine interventions regarding health outcomes, behavior changes, and engagement with health services. Nine databases were searched for studies that were randomized controlled trials in the English language, published before 4 December 2023. The searches identified 34 research articles that met the inclusion/exclusion criteria. This review revealed four key takeaways: (1) graphic medicine interventions are used globally; (2) graphic medicine interventions may be efficacious for a wide variety of health topics; (3) graphic medicine can be equitably delivered in many formats; and (4) graphic medicine can be applied broadly across the lifespan. The findings suggest that graphic medicine enhances patient engagement, empowers individuals with knowledge, and ultimately contributes to improved health outcomes across various populations; however, more effectiveness trials are needed. Additionally, an expanded definition of graphic medicine is presented.

## 1. Introduction

Health literacy is defined by the Centers for Disease Control and Prevention (CDC) as “the degree to which individuals have the ability to find, understand, and use information and services to inform health-related decisions and actions for themselves and others” [1]. With over one-third of Americans having low health literacy [2,3] health information is often difficult for the general public to understand [4]. Stossel et al. (2012) [5] found that the majority of patient education materials available at the point of care were written at reading levels considerably higher than that of the average American adult. Therefore, health communicators need better ways to inform patients and communities about health topics. Graphic medicine, which Czerwiec defines as “the intersection of the medium of comics and the discourse of health care”, presents opportunities to make information about health clearer and more approachable [6]. Despite graphic medicine’s potential to improve health education, little research has investigated its effectiveness in improving health outcomes and behaviors.

### What Is Graphic Medicine?

Graphic medicine tells stories using a combination of text and visuals, often employing comic components such as panels, images, speech, and thought balloons [7]. The National Library of Medicine defines graphic medicine as “the use of comics to tell stories of illness and health” [8]. By pairing illustrations with text, graphic medicine can provide an approachable medium for presenting complex health experiences that can be used by peers and healthcare providers to promote effective understanding. A seminal systematic review of the literature on the use of illustrations combined with text in health messaging found that such techniques improve attention to, and the recall of, health information, facilitate comprehension of the message, and influence intentions to act [9]. Given its benefits, graphic medicine is applicable across a variety of sectors, including public health, medicine, and education [10]. For example, graphic medicine can be used in medical education to boost empathy and improve communication skills among resident physicians [11,12]. In a qualitative survey of resident physicians participating in a novel curriculum featuring graphic medicine, 97% of residents reported the graphic medicine sessions to be a good use of their time [12]. In addition, graphic medicine can be helpful for patient education in hospital settings, particularly when explaining unfamiliar or anxiety-inducing concepts like surgery [13,14].

Although comics are often thought of as a medium for children, graphic medicine may be beneficial for both children and adults. For children, the benefit of illustrations combined with text in health education is well studied: one randomized study of 364 children found that story-format materials significantly improved comprehension compared to standard text-format materials [13]. In a more recent systematic review, graphic methods for achieving pediatric outcomes were explored, and pictorial images were found to be a preferred method when working with children who struggle with reading and text-based reporting measures [14]. In adults, health information presented with comics has also proven to be more informative than standard materials [14]. In a randomized trial comparing the efficacy of two vaccine information flyers, the comic flyer had a statistically significant effect on participants’ attitudes and perceptions of the flyer’s informativeness compared to the control flyer developed by the Centers for Disease Control and Prevention (CDC) [14].

Graphic medicine can be especially useful for providing health education for disenfranchised populations. The levels of health literacy are lower across many marginalized groups, including minoritized people, people without a high school degree, and people living in poverty [3,15]. Moreover, people with low health literacy are more likely to have poor health [16]. By increasing the accessibility of health information, graphic medicine can benefit diverse and marginalized populations [17]. Graphic medicine can also play a role in destigmatizing certain topics that may be difficult for people to discuss. For instance, a growing body of comics explores areas such as mental health [18], complex trauma [19], overdose response [20], death and dying [21], and HIV/AIDS [22].

Despite the ability of graphic medicine to improve understanding and reduce stigma, little research has investigated the effectiveness of these interventions in improving health. Thus, we conducted a scoping review to answer the following questions:

1. What is graphic medicine?
2. What are the modes of delivery often used in graphic medicine interventions?
3. What population groups have graphic medicine interventions been designed with and for?
4. What are the efficacy and/or effectiveness of graphic medicine interventions regarding health outcomes, behavior changes, or improved engagement with health services/care?

## 2. Materials and Methods

The search strategy, study selection, and data extraction were organized and are reported in accordance with the PRISMA extension for scoping reviews (PRISMA-ScR [23]), and this review is registered with the PROSPERO international prospective register of systematic reviews (CRD42022384477).

### 2.1. Inclusion/Exclusion Criteria

This review included experimental studies with a comparison or control group published in peer-reviewed articles, in the English language, and on humans. Studies were sought that reported on the efficacy or effectiveness of a graphic medicine intervention regarding behavioral changes (health self-management behaviors, coping behaviors, preventative behaviors, etc.); health outcomes (physical or mental); and health service engagement (accessing care, seeking care, obtaining primary care, attending treatment, continued engagement in treatment). Graphic medicine was defined as still imagery (not videos or video games) that promotes health education. No limit was put on the publication date. Records were excluded if they were gray literature, dissertations, or if the graphic medicine intervention was part of a larger multicomponent intervention where the effects could not be parsed out.

### 2.2. Literature Search Strategy

A comprehensive literature search strategy was developed and conducted by an experienced medical librarian with input from the research team to identify relevant articles. Search terms were derived from a previously published scoping review on graphic medicine [24]. The searches combined controlled vocabulary supplemented with keywords related to the concept of graphic medicine (e.g., cartoon, comic book) and trial design (e.g., randomized, control). The draft strategies were peer-reviewed by another medical librarian and retested.

**Figure 1:**
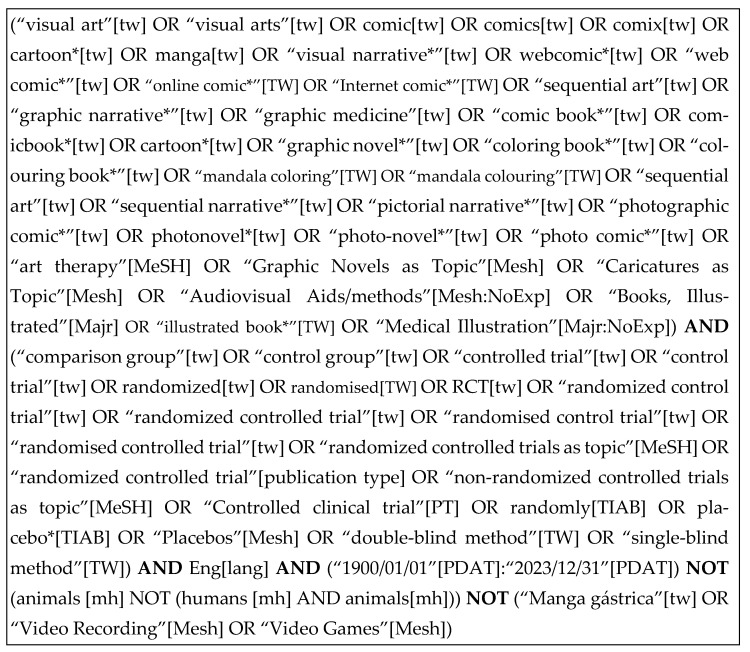
Search strategy for PubMed.

Searches were initially performed on 12 December 2022 and re-run for updates on 4 December 2023. Nine bibliographic databases were searched: APA PsycInfo (EBSCOhost); CINAHL (EBSCOhost); the Cochrane Database of Systematic Reviews; Embase.com; ERIC (EBSCOhost); Health Source: Nursing/Academic (EBSCOhost); LGBTQ+ Source (EBSCOhost); PubMed; and Scopus (Elsevier). The search strategy for PubMed may be found in Figure 1. Full search strategies for each database may be found in Appendix A.

### 2.3. Study Selection

**Figure 2:**
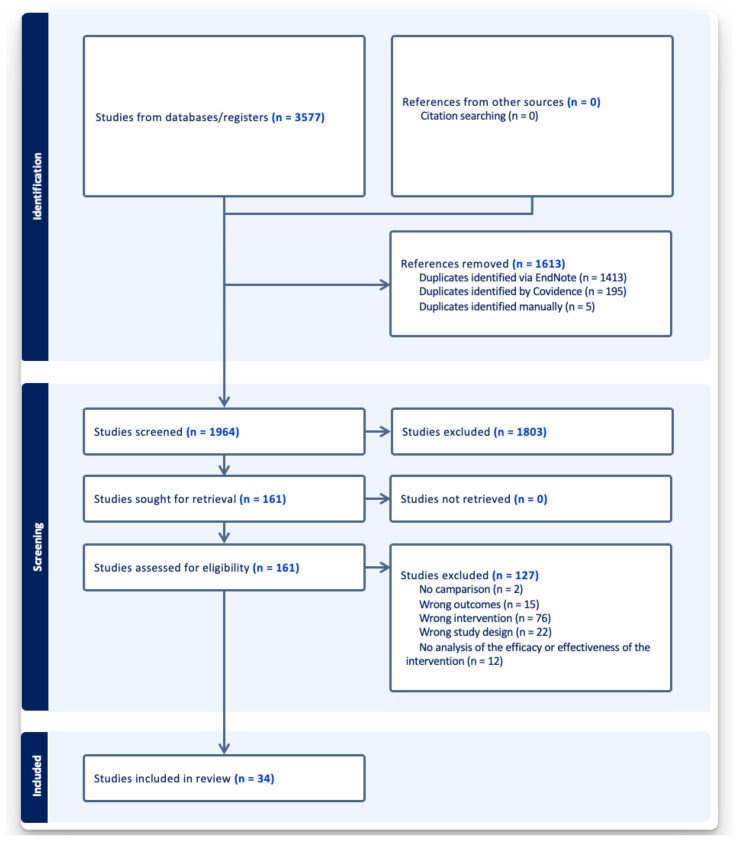
PRISMA 2020 flow diagram.

A total of 3577 citations from the database searches were uploaded to EndNote [25], which identified and excluded 1413 duplicates. This left 2164 studies, which were uploaded to the Covidence systematic review software platform [26]. Covidence identified 195 additional duplicates, and the study team identified 5 additional duplicates, leaving 1964 records. Title and abstract screening for eligibility was performed by two independent investigators according to the inclusion/exclusion criteria. Conflicts between the reviewers were resolved by a third reviewer. Of these records, 1803 were excluded for irrelevancy, leaving 161 eligible for a full-text review. Of these, 127 were excluded, leaving 34 for data extraction and synthesis. The review and selection processes for the studies are summarized in the diagram in Figure 2.

Additionally, the following websites were hand-searched: https://www.graphicmedicine.org/book-series/graphic-medicine-manifesto/ (accessed on 12 December 2022 and again for updates on 4 December 2023), https://www.nlm.nih.gov/exhibition/graphicmedicine/index.html, https://repository.escholarship.umassmed.edu/ (accessed on 12 December 2022 and again for updates on 4 December 2023), and https://www.psupress.org/books/series/book_SeriesGM.html (accessed on 12 December 2022 and again for updates on 4 December 2023), resulting in 0 additional articles for review.

### 2.4. Article Data Extraction and Synthesis

Data were extracted by two review authors and audited by a third author. Matrix tables were developed in Excel and Microsoft Word to extract data, which were then categorized by our two-member results team by our a priori outcomes (behavior changes, health, and health services used), populations, and interventions, as recommended by Maxwell (1996) [27]. As these categories were developed, we began a deductive and inductive analysis using an iterative process to describe and interpret our results via descriptive content analysis [28].

## 3. Results

To assess and compare the various graphic medicine articles included in this review, we extracted information on the author, year, purpose, research design, total number of participants, population of interest, outcomes of interest, timepoints, intervention and comparison groups, and key findings (see Table A1 in Appendix A.2). In line with the inclusion criteria, all 34 articles employed a randomized control trial (RCT) research design, with some classified more explicitly as quasi-experimental (*n* = 2), cluster randomized controlled trials (*n* = 1), or non-inferiority controlled trials (*n* = 1). As intended, all articles included in this scoping review reported on the efficacy or effectiveness of a graphic medicine intervention regarding either behavioral changes, health outcomes, or health service engagement. The following results are presented to address our research questions: What is graphic medicine? What are the modes of delivery often used in graphic medicine interventions? What population groups have graphic medicine interventions been designed with and for? What are the efficacy and/or the effectiveness of graphic medicine interventions regarding health outcomes, behavior changes, or improved engagement with health services/care?

### 3.1. Defining Graphic Medicine

**Figure 3:**
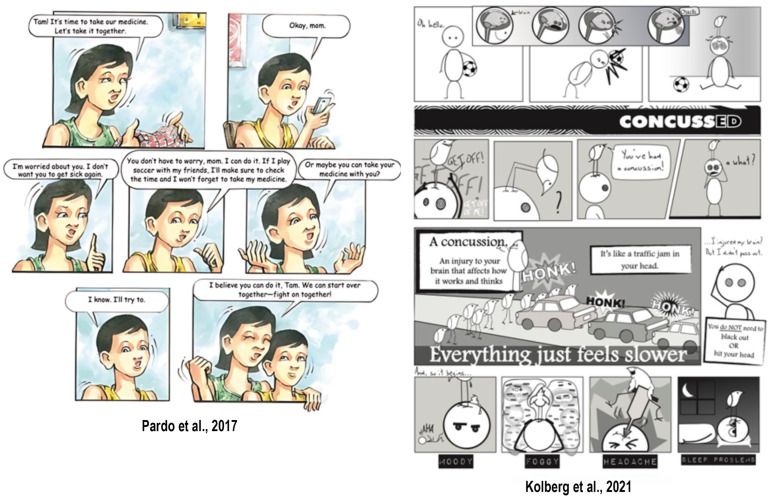
Example images of comic-style graphic medicine interventions [33,41].

**Figure 4:**
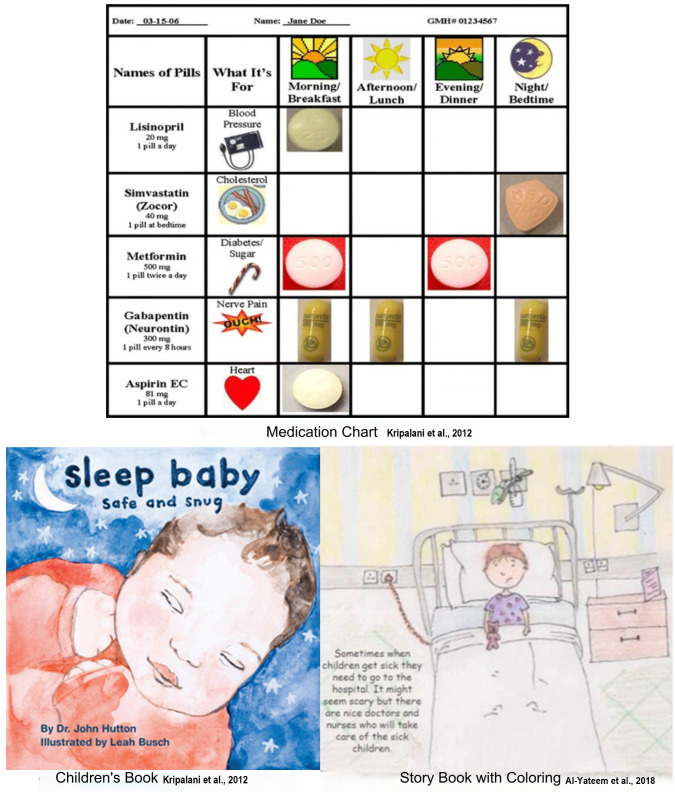
Example images of non-comic-style graphic medicine interventions [47,51,54].

We found that in the context of healthcare, graphic medicine is a versatile medium that is not easily defined. From the use of graphic elements to sequential comics, graphic medicine was exemplified in multiple forms (Table A1). Traditional narrative and sequential comics were used throughout the studies to convey health-related information, engage readers, and facilitate learning. Authors often referred to comics using different descriptors: comics [29,30], comic leaflets [31,32], educational comics [33,34], comic books [35,36,37,38], cartoon pictures [39], cartoons [40,41], brochures with cartoon images [42], and manga [43,44,45] (Figure 3). Products that were non-sequential or lacked narrative linking using graphics included the following: illustrated books [46,47,48], printed materials [49], pictographs [50], children’s books/brochures [51], education books [52], pictorial aids [53], stories and coloring activities [54], pictorial images [55], graphic narratives [56], and educational cartoons [57] (Figure 4).

As a result of these findings, our augmented definition of graphic medicine is as follows:

Graphic medicine is a versatile medium that employs visual storytelling techniques, such as comics, graphic novels, illustrations, and cartoons, to convey medical information, educate patients about their conditions, improve treatment adherence, and facilitate communication between patients and healthcare providers. It encompasses a wide range of formats, including traditional narrative comics, manga-style illustrations and formats (read from the traditional back to the front), and graphic aids, each uniquely suited to addressing various healthcare needs and behavior change goals across different stages of life, from youth to end-of-life care. Graphic medicine interventions are designed to be engaging, understandable, and culturally relevant, potentially reaching diverse populations and promoting health equity by overcoming barriers such as low health literacy, language barriers, and disabilities affecting verbal communication. Through colorful illustrations, relatable characters, and age-appropriate language, graphic medicine interventions aim to alleviate anxiety and distress, enhance the understanding of medical concepts, promote adherence to treatment regimens, and facilitate better communication between patients, caregivers, and healthcare providers.

### 3.2. Graphic Medicine Design Processes and Modes of Delivery

Most developers began with a thorough literature review to ensure evidence-based content and an understanding of the existing interventions. The amount of community or end user involvement in the design process varied among the included articles. However, many studies intentionally engaged numerous key informants—such as educators [36], students, healthcare professionals [47], end users, and end user support people (e.g., parents, spouses, etc. [14,39,41,51])—to ensure that the content and format effectively addressed the needs and preferences of the target audience. Several research teams used an iterative process, which included several step-wise examinations of the developing interventions to ensure the content was relevant and acceptable to end users [38] and for those with disabilities such as hearing impairment [46], and significant community engagement, considering the demographic characteristics and needs of the low-socioeconomic-status population [51]. A simplistic intervention design was found in Kripalani et al. (2012) [47], wherein clip art such as pictures of eggs and bacon in a calendar format was utilized, indicating minimal effort and thought in the design of the educational materials. Some studies had limited engagement with communities and/or end users and other key informants [13,40,58]. However, other studies demonstrated a strong commitment to community consultation [58], the incorporation of literature reviews [49], interdisciplinary collaboration [33], and cultural adaptations [40].

The modes of delivery varied widely, often depending on the material and target audience. For instance, several articles used digital delivery. Imamura et al. (2014) [43] opted for an online approach, offering participants a series of stress management lessons delivered through a manga comic format. Other articles described in-person delivery with accompanying one-to-one counseling/instruction. Garcia de Avila et al. (2022) [49] paired an educational comic with a preoperative guidance session delivered by nurses, which included verbal information combined with the comic book. Some interventions also featured interactive components to enhance the learning and retention of health-related information [34,35,37,41]. While these interventions shared common characteristics, variations may have existed in the specific topics covered, format, and delivery methods used, influenced by cultural and intervention contexts and making a formal meta-analysis difficult [32,34,40,58].

### 3.3. The Application of Graphic Medicine Across the Lifespan

**Figure 5:**
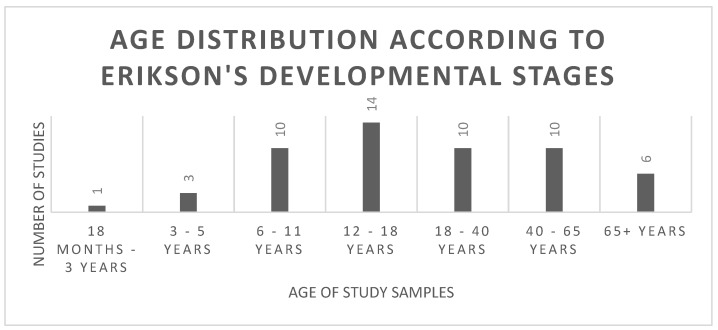
Age distribution according to Erikson’s Developmental Stages.

Graphic medicine can be used across the life course, and this was reflected in the included articles. We present the age distribution according to the stages of development according to Erikson as this theory focuses on development across the lifespan [59]. Seven of the eight stages were identified in this review (Figure 5).

#### 3.3.1. Youth

A graphic medicine approach provides a unique platform to address the healthcare needs of children and adolescents, presenting complex medical information in visually appealing and accessible formats. Interventions using graphic medicine for youth typically incorporate visually appealing elements such as colorful illustrations and relatable characters to capture the attention and interest of children and adolescents. Tailored to the age and developmental stage of the target audience, the content of these interventions addresses various medical topics, including those related to health conditions, such as safe sleep education, the prevention and management of low back pain, concussion recognition, the prevention and management of epilepsy, medical procedures, treatment regimens, and preventive care measures [33,34,35,36,51]. Emotional engagement is often incorporated to connect with youth on a deeper level, addressing feelings of anxiety [29,31,32,49,60], distress [43,52], or fear related to medical experiences, while portraying positive outcomes and coping strategies [32,45,48,54]. Numerous studies, including those by Kassai et al. (2016) [31], Kolberg et al. (2021) [33], and Nestadt et al. (2019) [41], have demonstrated graphic medicine’s efficacy in improving health outcomes among youth. These interventions have been shown to reduce anxiety and depression, enhance the understanding of medical concepts, promote adherence to treatment regimens, and facilitate better communication between patients, caregivers, and healthcare providers. Additionally, graphic medicine was used in Lusiana et al. (2023) [30] to influence behavior changes aimed at improving the nutritional knowledge and behavior of elementary school students.

#### 3.3.2. Adults

Having demonstrated its versatility and potential to improve health outcomes among youth, graphic medicine seamlessly transitions into adult healthcare, where its multi-faceted roles become increasingly evident. For instance, Brand et al. (2019) [29] explored its impact on patient comprehension and anxiety reduction before coronary procedures, suggesting its efficacy in alleviating anxiety and enhancing understanding among adults facing medical interventions. In a familial context, Bhana et al. (2004) [40] demonstrated how graphic medicine facilitates communication about sensitive topics within families, employing cartoons and narratives to ease discussions on issues like AIDS transmission and stigma among parents and pre-adolescent children. Similarly, Bazzano et al. (2023) [60] demonstrated how graphic medicine effectively addresses the needs of adults, particularly in the context of healthcare anxiety. By using a graphic novel as an intervention for patients awaiting oral biopsies, the study illustrated the power of visual storytelling in conveying complex medical information and alleviating anxiety. Through colorful vignettes depicting the biopsy procedure and its aftermath, the graphic novel provided patients with a visual narrative that enhanced their understanding of the process while also mitigating their fears and anxieties. This approach catered to adults by acknowledging their cognitive abilities and emotional experiences, offering a unique blend of information dissemination and emotional support that was accessible and engaging.

#### 3.3.3. End of Life

As individuals progress through the lifespan, graphic medicine continues to be relevant in end-of-life care. Studies like that by Ke et al. (2021) [42] emphasize its importance in facilitating communication and understanding between older patients and their surrogates regarding end-of-life care preferences. By providing a visual medium to explore themes of mortality and grief, graphic narratives help bridge the communication gap between patients and their families, ensuring that end-of-life care preferences are aligned. Evidence from these studies underscores the broad applicability of graphic medicine in addressing healthcare needs and behavior change goals across different stages of life, from youth to end-of-life care.

### 3.4. Application of Graphic Medicine Among Diverse Topics and Populations and Health Equity

In addition to its broad applicability across different stages of life, graphic medicine serves as a versatile tool for addressing a multitude of health-related issues among varying populations. Zhou et al. (2023) [61] and Shin et al. (2022) [38] demonstrated the application of graphic medicine among ethnically and racially diverse populations, specifically targeting Mexican American women and East African Americans, respectively. These studies placed heavy emphasis on using culturally relevant narrative messages to reduce health disparities associated with various behaviors, thereby promoting health equity. Graphic medicine is uniquely positioned to increase understanding and acceptability among those with low literacy levels, language barriers, or disabilities that may affect verbal communication [33,46,55]. By using such visuals, one can bridge communication gaps and ensure that important information is effectively conveyed to all end users [38,41,53,55,61], regardless of their individual characteristics or circumstances.

An intervention by Arunakul et al. (2012) [46] was specifically designed for hearing-impaired children aged 6–10, considering their communication needs and learning abilities. The use of sign language and clear illustrations in an illustrated book catered to the unique requirements of this population. Thus, using visual aids and sign language may have facilitated the better comprehension and retention of oral health information among participants. Moreover, sleep problems are common in children with learning disabilities, but effective interventions are scarce. Montgomery et al. (2004) [48] aimed to address sleep problems in learning-disabled children aged 2–8, which are often prevalent and challenging to treat. Additionally, the booklet was designed to be easy to understand, making it accessible to parents with varying literacy levels. This approach aimed to overcome barriers to accessing traditional face-to-face treatments, such as limited resources and professional expertise. By providing accessible and convenient interventions, this approach could potentially improve the quality of life for both children and their families.

### 3.5. Efficacy and Effectiveness of Graphic Medicine Interventions

A total of *n* = 34 graphic medicine studies were identified for this review and spanned countries across five of the six inhabited continents. These studies employed a variety of randomized controlled trial (RCT) designs, including randomized non-inferiority controlled trials [54], cluster randomized controlled trials [35], and quasi-experimental designs [30,42], among others. See Table A1 in Appendix A.2. This review illuminated mixed findings on the efficacy of graphic medicine and, although studies may have claimed to report on effectiveness trials, only one effectiveness study was identified, making it difficult to report on the effectiveness of graphic medicine as an intervention medium. The populations, methods, sample sizes, and control groups varied. Kulkarni et al. (2022) [32] sought to reduce preoperative anxiety in children aged 6–12 in an efficacy trial with a comic leaflet intervention with oral instructions compared to oral instructions alone (control). This study reported no significant reduction in preoperative anxiety compared to the control group. Conversely, in a proof-of-concept pilot study of preprocedural anxiety, Brand et al. (2019) [29] compared an informed consent standard (control) to informed consent with a comic supplement, finding a significant reduction in anxiety in the comic book group (*p* < 0.001). Similarly, infant sleep knowledge improved among mothers with a low socioeconomic status in the efficacy trial ‘Sleepy Baby: Safe and Snug’ [51]. Sleep knowledge was improved in both the ‘Sleepy Baby: Safe and Snug’ intervention and control (brochure) groups, while the intervention led to the behavior modifications of less bed sharing and exclusive crib use [51]. Kripalani et al. (2012) [47] aimed to test the efficacy of an illustrated medication schedule for medication adherence. No significant differences were found compared to controls. Simple graphics were added to calendars to enhance health literacy (e.g., bacon and eggs to remind someone to take a statin). Although adherence was not statistically improved in that study, adherence to antiretroviral therapy among adolescents in Thailand improved after a different, culturally tailored, cartoon-based comic intervention (11 sessions) was delivered over six and nine months compared to standard care [41].

Lastly, Al-Yateem et al. (2018) [54] conducted the only effectiveness study in our sample. The non-inferiority trial of a graphic medicine coloring book play and distraction intervention, “Adam Goes to Surgery”, versus preoperative medication (control group) incorporated narrative storytelling to reduce anxiety among children during perioperative day surgery. The graphic intervention was multilayered as a distraction tool and included a narrative, which introduced equipment, personnel, and procedures, combined with coloring. The book was also read to the children by their parents, allowing them time to answer questions. However, the differences were not significant enough to prove superiority (modified Yale Preoperative Assessment Scale (MyPAS) (*p* = 0.941), State-Trait Anxiety Inventory for Children (*p* = 0.708)).

## 4. Discussion

This review of graphic medicine interventions revealed four key takeaways: (1) graphic medicine interventions are used globally; (2) graphic medicine interventions may be efficacious and effective regarding a wide variety of health-related topics; (3) graphic medicine can be equitably delivered in many formats; and (4) graphic medicine can be applied broadly across the lifespan. Historically, graphic medicine was rooted in comic book culture, and this review captured some of this history with the inclusion of Gillies et al. (1990) [58], which used a volume of Streetwize UK, a comic published in the 1980s and 1990s which was co-created with community members at the intersection of art and health. Since that time, graphic medicine interventions have spread across the globe. Despite our review criteria only including articles published in English, the included articles represented RCTs conducted in five of the six inhabited continents, with broad applications for a range of health topics. More specifically, graphic medicine interventions have been shown to be efficacious and effective in areas such as mental health, behavioral dynamics, chronic conditions, health promotion, and sensitive topics such as HIV/AIDS [35,41,43,53,58,60].

The studies in this review illustrate how graphic medicine interventions have been employed to tackle various health concerns among diverse groups of people and communities. From reducing anxiety before coronary procedures to improving mental health literacy and medication adherence to changing lifestyle habits, graphic medicine interventions have proven instrumental in enhancing patient understanding, promoting behavioral change, and facilitating better health outcomes. The included articles highlight the potential of graphic medicine to address health disparities and promote health equity by using culturally relevant and visually appealing mediums to deliver health information. This breadth of applications underscores the significance of graphic medicine as a versatile tool in modern healthcare, capable of addressing diverse health issues and empowering individuals across different stages of life.

Although graphic medicine is often considered especially useful and particularly appealing to children and adolescents [62], these interventions can be applied across the lifespan [63]. Graphic medicine and visual storytelling can facilitate the delivery of complex medical information in a format that is accessible and more easily digestible than traditional formats [64,65]. In turn, this can help reinforce health education for children and adults. The use of comics in the delivery of health information can have a normalizing effect, increasing feelings of hopefulness and decreasing feelings of isolation among people experiencing physical or mental illness, as well as those living with marginalized social identities [66,67,68].

### 4.1. Strengths and Limitations

There are several strengths of this scoping review, including dual-reviewer systematic article identification, dual-reviewer data extraction, and the systematic auditing of the extracted data and data synthesis. There are also limitations of the current review, including potentially missed articles due to the exclusion of gray material and dissertations, single-reviewer article quality appraisal, and the narrow scope of particular health-related outcomes. Due to the broad nature of the inclusion criteria, which selected for (1) experimental, (2) human-centered (3) studies in the English language (4) with a comparison or control group, (5) published in peer-reviewed articles, this scoping review covers articles on a variety of graphic medicine interventions. All interventions, though different in form, met this review’s definition of graphic medicine: non-video, still imagery that promotes health education. However, despite meeting this criterion, many of the included graphic medicine interventions were fundamentally different. For instance, we recognize that manga and illustrated children’s books differ greatly in their target audience, level of sophistication, and overall structure. On the one hand, including a wide range of graphic medicine interventions may have made it difficult to draw conclusions about any one form of imagery or mode of delivery. On the other hand, this variation between articles is part of the scoping review process, and we intentionally employed broad inclusion criteria to capture a large number of peer-reviewed articles and to investigate the field of graphic medicine as a whole without favoring any one type of imagery or mode of delivery. Despite these limitations, this review provides a meaningful synthesis of the current state of the graphic medicine intervention RCT literature, which can be used to guide future intervention development and adaptation for diverse populations.

### 4.2. The Way Forward: Implications and Recommendations

Graphic medicine has been established as a novel source of diverse graphic narratives in varied contexts and should be broadly implemented across health disciplines. It has been nearly a decade since six pioneers of the field published the Graphic Medicine Manifesto [6]. This collection of essays stressed the need for diverse and interdisciplinary perspectives, inclusive of healthcare workers, patients, and educators. Although there are notable graphic medicine novels and related research articles across health disciplines, the formal integration of this topic has been fractured. Graphic medicine has notably been implemented in health curricula largely for medical students, particularly for boosting empathy and communication skills [12]. Graphic medicine could also make a significant impact in the nursing profession. As the largest segment of the healthcare workforce [69] and with a worsening nursing shortage in America [70] and globally [71], graphic medicine could be used as an innovative supplement to traditional health education. This may have positive effects on retention in training programs and may assist clinicians in delivering health content to patients, further supporting their retention in clinical care and improve patient knowledge, intention, and behaviors.

Recently, the Coronavirus Disease 2019 (COVID-19) pandemic represented a turning point in the scale of the use of graphic medicine to deliver health-related instructional, personal, and therapeutic content, largely via social media [72]. In 2020, graphic medicine became an important medium for alerting the public to the nature of the pandemic as well as for sharing pertinent information about transmissibility and safety. Graphics were created by the world’s most influential health organizations (e.g., CDC, WHO) and shared across social media platforms [72]. Consequently, globally, people of all ages, including older adults [63], vastly expanded their social media engagement. While the graphics and visual storytelling may have improved COVID-19-related health literacy, there were unintended consequences as the creation and dissemination of graphic medicine content became unregulated, and misinformation was shared by various sources [73]. This is a major concern for clinicians and public health officials moving forward, emphasizing the need for evidence-based and community-co-created graphic medicine and public education regarding how to identify a trusted source of health information.

This review highlights four key insights: (1) graphic medicine interventions are implemented worldwide; (2) they show potential efficacy across a broad range of health topics; (3) they can be delivered equitably through diverse formats; and (4) they are applicable across all stages of life. While the findings suggest that graphic medicine fosters patient engagement, supports knowledge empowerment, and may lead to improved health outcomes across diverse populations, more effectiveness trials are necessary to strengthen the evidence base.

## 5. Conclusions

With their highly accessible format, graphic medicine interventions show promising efficacy in improving health outcomes through patient engagement and knowledge empowerment. While further effectiveness trials are necessary to strengthen the evidence base, they have the potential to benefit diverse communities across a range of health topics throughout the lifespan through the use of culturally relevant materials in a variety of formats.

## Appendix A

### Appendix A.1. Full Literature Search Strategies

**Databases and Search Strategies** **All Searches Run in Firefox Browser**	**Records Retrieved on** **12/12/22**	**Records Retrieved on 12/4/23**
APA PsycInfo (EBSCOhost) Apply equivalent subjects unchecked AB(“visual art” OR “visual arts” OR comic OR comics OR comix OR cartoon* OR manga OR “visual narrative*” OR webcomic* OR “web comic*” OR “online comic*” OR “Internet comic*” OR “sequential art” OR “graphic narrative*” OR “graphic medicine” OR “comic book*” OR comicbook* OR cartoon* OR “graphic novel*” OR “coloring book*” OR “colouring book*” OR “mandala coloring” OR “mandala colouring” OR “sequential art” OR “sequential narrative*” OR “pictorial narrative*” OR “photographic comic*” OR photonovel* OR “photo-novel*” OR “photo comic*” OR “art therapy” OR “illustrated book*” OR “medical illustration”) AND (DE “Randomized Clinical Trials” OR “comparison group” OR “control group” OR “controlled trial” OR “control trial” OR randomized OR RCT OR “randomized control trial” OR “randomized controlled trial” OR “randomised control trial” OR “randomised controlled trial” OR “Controlled clinical trial” OR random* OR placebo* OR “double-blind method” OR “single-blind method”) Limits—Population Group: Human; Document Type: Journal Article	364	18
Cochrane Database of Systematic Reviews (“visual art” OR “visual arts” OR comic OR comics OR comix OR cartoon* OR manga OR “visual narrative*” OR webcomic* OR “web comic*” OR “online comic*” OR “Internet comic*” OR “sequential art” OR “graphic narrative*” OR webcomic* OR “web comic*” OR “online comic*” OR “Internet comic*” OR “sequential art” OR “graphic narrative*” OR “graphic medicine” OR “comic book*” OR comicbook* OR cartoon* OR “graphic novel*” OR “coloring book*” OR “colouring book*” OR “mandala coloring” OR “mandala colouring” OR “sequential art” OR “sequential narrative*” OR “pictorial narrative*” OR “photographic comic*” OR photonovel* OR “photo-novel*” OR “photo comic*” OR “art therapy” OR “illustrated book*” OR “medical illustration”)	25	1
CINAHL (EBSCOhost) Apply equivalent subjects unchecked (MH “Graphic Medicine” OR “visual art” OR “visual arts” OR comic OR comics OR comix OR cartoon* OR manga OR “visual narrative*” OR webcomic* OR “web comic*” OR “online comic*” OR “Internet comic*” OR “sequential art” OR “graphic narrative*” OR “graphic medicine” OR “comic book*” OR comicbook* OR cartoon* OR “graphic novel*” OR “coloring book*” OR “colouring book*” OR “mandala coloring” OR “mandala colouring” OR “sequential art” OR “sequential narrative*” OR “pictorial narrative*” OR “photographic comic*” OR photonovel* OR “photo-novel*” OR “photo comic*” OR “art therapy” OR “illustrated book*” OR “medical illustration”) **AND** (MH “Randomized Controlled Trials” OR “comparison group” OR “control group” OR “controlled trial” OR “control trial” OR randomized OR RCT OR “randomized control trial” OR “randomized controlled trial” OR “randomised control trial” OR “randomised controlled trial” OR “Controlled clinical trial” OR random* OR placebo* OR “double-blind method” OR “single-blind method”) Limits—Human; Publication Type: Journal Article; Language: English	262	29
Embase.com (mapping turned off) (‘art’/exp OR ‘visual art’ OR ‘visual arts’ OR comic OR comics OR comix OR manga OR ‘visual narrative*’ OR webcomic* OR ‘web comic*’ OR ‘online comic*’ OR ‘internet comic*’ OR ‘graphic narrative*’ OR ‘graphic medicine’ OR ‘comic book*’ OR comicbook* OR cartoon* OR ‘graphic novel*’ OR ‘coloring book*’ OR ‘colouring book*’ OR ‘mandala coloring’ OR ‘mandala colouring’ OR ‘sequential art’ OR ‘sequential narrative*’ OR ‘pictorial narrative*’ OR ‘photographic comic*’ OR photonovel* OR ‘photo-novel*’ OR ‘photo comic*’ OR ‘art therapy’ OR ‘illustrated book*’ OR ‘medical illustration’) **AND** (‘comparison group’ OR ‘control group’ OR ‘controlled trial’ OR ‘control trial’ OR randomized OR rct OR ‘randomized control trial’ OR ‘randomized controlled trial’ OR ‘randomised control trial’ OR ‘randomised controlled trial’ OR ‘controlled clinical trial’ OR random* OR placebo* OR ‘double-blind method’ OR ‘single-blind method’) **AND** ([randomized controlled trial]/lim OR ‘controlled clinical trial’/de) **AND** [article]/lim **AND** [english]/lim **AND** [humans]/lim **NOT** ‘video game’/exp	646	165
ERIC (EBSCOhost) Apply equivalent subjects unchecked (DE “Cartoons” OR “visual art” OR “visual arts” OR comic OR comics OR comix OR cartoon* OR manga OR “visual narrative*” OR webcomic* OR “web comic*” OR “online comic*” OR “Internet comic*” OR “sequential art” OR “graphic narrative*” OR “graphic medicine” OR “comic book*” OR comicbook* OR cartoon* OR “graphic novel*” OR “coloring book*” OR “colouring book*” OR “mandala coloring” OR “mandala colouring” OR “sequential art” OR “sequential narrative*” OR “pictorial narrative*” OR “photographic comic*” OR photonovel* OR “photo-novel*” OR “photo comic*” OR “art therapy” OR “illustrated book*” OR “medical illustration”) **AND** (DE “Randomized Controlled Trials” OR “comparison group” OR “control group” OR “controlled trial” OR “control trial” OR randomized OR RCT OR “randomized control trial” OR “randomized controlled trial” OR “randomised control trial” OR “randomised controlled trial” OR “Controlled clinical trial” OR random* OR placebo* OR “double-blind method” OR “single-blind method”) Limits—Publication Type: Journal Articles; Language: English	172	7
Health Source: Nursing/Academic (EBSCOhost) Apply equivalent subjects unchecked (DE “GRAPHIC novels” OR “visual art” OR “visual arts” OR comic OR comics OR comix OR cartoon* OR manga OR “visual narrative*” OR webcomic* OR “web comic*” OR “online comic*” OR “Internet comic*” OR “sequential art” OR “graphic narrative*” OR “graphic medicine” OR “comic book*” OR comicbook* OR cartoon* OR “graphic novel*” OR “coloring book*” OR “colouring book*” OR “mandala coloring” OR “mandala colouring” OR “sequential art” OR “sequential narrative*” OR “pictorial narrative*” OR “photographic comic*” OR photonovel* OR “photo-novel*” OR “photo comic*” OR “art therapy” OR “illustrated book*” OR “medical illustration”) **AND** (DE “RANDOMIZED controlled trials” OR “comparison group” OR “control group” OR “controlled trial” OR “control trial” OR randomized OR RCT OR “randomized control trial” OR “randomized controlled trial” OR “randomised control trial” OR “randomised controlled trial” OR “Controlled clinical trial” OR random* OR placebo* OR “double-blind method” OR “single-blind method”) Limits—Document Type: Article	109	9
LGBTQ+ Source (EBSCOhost) Apply equivalent subjects unchecked (DE “GRAPHIC novels” OR “visual art” OR “visual arts” OR comic OR comics OR comix OR cartoon* OR manga OR “visual narrative*” OR webcomic* OR “web comic*” OR “online comic*” OR “Internet comic*” OR “sequential art” OR “graphic narrative*” OR “graphic medicine” OR “comic book*” OR comicbook* OR cartoon* OR “graphic novel*” OR “coloring book*” OR “colouring book*” OR “mandala coloring” OR “mandala colouring” OR “sequential art” OR “sequential narrative*” OR “pictorial narrative*” OR “photographic comic*” OR photonovel* OR “photo-novel*” OR “photo comic*” OR “art therapy” OR “illustrated book*” OR “medical illustration”) **AND** (DE “CLINICAL trials” OR “comparison group” OR “control group” OR “controlled trial” OR “control trial” OR randomized OR RCT OR “randomized control trial” OR “randomized controlled trial” OR “randomised control trial” OR “randomised controlled trial” OR “Controlled clinical trial” OR random* OR placebo* OR “double-blind method” OR “single-blind method”) Limits—Document Type: Article	6	0
PubMed (“visual art”[tw] OR “visual arts”[tw] OR comic[tw] OR comics[tw] OR comix[tw] OR cartoon*[tw] OR manga[tw] OR “visual narrative*”[tw] OR webcomic*[tw] OR “web comic*”[tw] OR “online comic*”[TW] OR “Internet comic*”[TW] OR “sequential art”[tw] OR “graphic narrative*”[tw] OR “graphic medicine”[tw] OR “comic book*”[tw] OR comicbook*[tw] OR cartoon*[tw] OR “graphic novel*”[tw] OR “coloring book*”[tw] OR “colouring book*”[tw] OR “mandala coloring”[TW] OR “mandala colouring”[TW] OR “sequential art”[tw] OR “sequential narrative*”[tw] OR “pictorial narrative*”[tw] OR “photographic comic*”[tw] OR photonovel*[tw] OR “photo-novel*”[tw] OR “photo comic*”[tw] OR “art therapy”[MeSH] OR “Graphic Novels as Topic”[Mesh] OR “Caricatures as Topic”[Mesh] OR “Audiovisual Aids/methods”[Mesh:NoExp] OR “Books, Illustrated”[Majr] OR “illustrated book*”[TW] OR “Medical Illustration”[Majr:NoExp]) **AND** (“comparison group”[tw] OR “control group”[tw] OR “controlled trial”[tw] OR “control trial”[tw] OR randomized[tw] OR randomised[TW] OR RCT[tw] OR “randomized control trial”[tw] OR “randomized controlled trial”[tw] OR “randomised control trial”[tw] OR “randomised controlled trial”[tw] OR “randomized controlled trials as topic”[MeSH] OR “randomized controlled trial”[publication type] OR “non-randomized controlled trials as topic”[MeSH] OR “Controlled clinical trial”[PT] OR randomly[TIAB] OR placebo*[TIAB] OR “Placebos”[Mesh] OR “double-blind method”[TW] OR “single-blind method”[TW]) **AND** Eng[lang] **NOT** (animals [mh] NOT (humans [mh] AND animals[mh])) **NOT** (“Manga gástrica”[tw] OR “Video Recording”[Mesh] OR “Video Games”[Mesh])	586	56
Scopus TITLE-ABS(“visual art” OR “visual arts” OR comic OR comics OR comix OR cartoon* OR manga OR “visual narrative*” OR webcomic* OR “web comic*” OR “online comic*” OR “Internet comic*” OR “sequential art” OR “graphic narrative*” OR “graphic medicine” OR “comic book*” OR comicbook* OR cartoon* OR “graphic novel*” OR “coloring book*” OR “colouring book*” OR “mandala coloring” OR “mandala colouring” OR “sequential art” OR “sequential narrative*” OR “pictorial narrative*” OR “photographic comic*” OR photonovel* OR “photo-novel*” OR “photo comic*” OR “art therapy” OR “illustrated book*” OR “medical illustration”) **AND** TITLE-ABS(“comparison group” OR “control group” OR “controlled trial” OR “control trial” OR randomized OR RCT OR “randomized control trial” OR “randomized controlled trial” OR “randomised control trial” OR “randomised controlled trial” OR “Controlled clinical trial” OR random* OR placebo* OR “double-blind method” OR “single-blind method”) **AND** (LIMIT-TO (LANGUAGE, “English”)) AND (LIMIT-TO (DOCTYPE, “ar”)) **AND NOT** “video games”	907	215
Totals		
Initial number of citations	3077	500
Duplicates deleted by Endnote	1271	142
Number uploaded to Covidence	1806	358
Duplicates deleted by Covidence	50	145
Number of citations screened	1756	213

### Appendix A.2. Description of Included Articles

**Table A1:** Description of included articles.

Author (Year), Country	Purpose	Research Design	Total Number of Participants, Population of Interest	Outcomes of Interest	Timepoints	Intervention and Comparison Groups	Key Findings
Al-Yateem et al. (2018) [54] United Arab Emirates and Jordan	To explore the efficiacy of play distraction compared to that of traditional pharmacological premedication in reducing anxiety levels in children undergoing day surgery.	Randomized control trial (RCT); non-inferiority trial	N = 168 Children aged 3–8 years undergoing elective day surgery	Health outcomes: anxiety levels, vital signs (heart rate [HR], respiratory rate, blood pressure)	Preoperative, intraoperative, postoperative	Intervention: Play distraction using a custom-made story and coloring book (“Adam Goes to Surgery”) Comparison: Pharmacological premedication (Midazolam)	Anxiety levels assessed via modified Yale Preoperative Assessment Scale (mYPAS): No significant difference between groups (mean score of 10.95 vs. 10.94, 95% CI [−0.35, 0.37], *p* = 0.941). State-Trait Anxiety Inventory for Children (STAIC) (parent-reported): Mean scores similar between groups (20.90 vs. 20.73, 95% CI [−0.52, 0.88], *p* = 0.708). Vital signs: No significant differences in HR, respiratory rate, or blood pressure at any timepoint between groups.
Arunakul et al. (2012) [46] Thailand	To evaluate the effectiveness of oral hygiene instruction media in influencing periodontal health among hearing-impaired children.	RCT	N = 80 (66 completed post-exam) Hearing-impaired children aged 6–10 years old	Health outcomes: oral hygiene status, plaque index (PI), gingival bleeding index (BI), and gingival index (GI)	Baseline, three months post-intervention	Intervention: Video presentation, illustrated book, video + illustrated book All received professional prophylaxis fluoride until plaque score = 0 at baseline Comparison: Control group with no additional media intervention All received professional prophylaxis fluoride until plaque score = 0 at baseline	All groups showed a significant reduction in their plaque scores, gingival bleeding index, and gingival index (*p* < 0.01) at three months. There was no statistically significant difference in periodontal health outcomes between the media types and control.
Bazzano et al. (2023) [60] Palermo, Italy	To study the effect of graphic novels in reducing the anxiety of patients waiting for an incisional biopsy in an oral oncology setting.	RCT	N = 50 Patients with clinical suspicion of oral potentially malignant disorders	Health outcomes: anxiety levels before an oral biopsy	Baseline, post-evaluation	Intervention: 25 patients who were given a graphic novel to read while waiting for the biopsy Comparison: 25 patients who did not receive the graphic novel and served as the control group	Beck Depression Inventory (BDI) Scores: Control Group: mean ± SD = 13.48 ± 10.29. Intervention Group: mean ± SD = 6.24 ± 6.11. Depression, Anxiety and Stress Scale (DASS-21) Scores: Control Group: mean ± SD = 16.4 ± 7.55. Intervention Group: mean ± SD = 6.08 ± 4.62. Statistical Significance: The graphic novel significantly improved the ability of the test group to tolerate anxiety while waiting for the oral biopsy, with significant differences observed in both the BDI and DASS-21 scores compared to the control group (*p* < 0.05).
Bechi et al. (2015) [39] Italy	To compare the efficacy of two social cognitive interventions (using videotaped material and comic strips) combined with domain-specific Cognitive Remediation Therapy (CRT) regarding the Theory of Mind (ToM) and daily functioning in schizophrenia patients.	RCT	N = 75 Patients with schizophrenia treated with CRT	Improvement in ToM and social cognitive abilities	Baseline; 12, 16, and 18 weeks post-intervention	Intervention: Social Cognitive Training (SCT) using videotaped materials and Theory of Mind Intervention (ToMI) using comic strips Comparison: Active control group using newspaper discussion and CRT	Both SCT and ToMI significantly improved ToM scores (*p* < 0.05), with no significant differences between two intervention groups. Improvements in ToM were not influenced by neuropsychological domains.
Bhana et al. (2004) [40] South Africa	To adapt and pilot the CHAMP (Collaborative HIV/AIDS and Adolescent Mental Health Program) in South Africa, focusing on outcome effects among adults.	RCT; quasi-experimental pre–post design	N = 124 families (mothers in the family) South African families with pre-adolescent children (10–11 years) in urban and semi-rural areas	Behavioral change outcomes (e.g., health self-management behaviors, coping behaviors); health outcomes (e.g., attitude changes and increased knowledge about AIDS)	Pre–post (after five intervention session)	Intervention: CHAMP intervention modified for families in South Africa using cartoon-based storyline No comparison	AIDS Transmission Knowledge: Significant improvement in the intervention group compared to the control group (F_1,164_ = 9.42, *p* < 0.003). AIDS Myth Knowledge: Significant improvement in the intervention group compared to the control group (F_1,166_ = 82.15, *p* < 0.001). Stigma: No significant differences between groups but a trend towards less stigmatizing attitudes in the intervention group. Parental Communication Styles: Significant shift towards more assertive communication in the intervention group (F_1,164_ = 7.40, *p* < 0.01). Hard to Talk About Topics: Significant improvement in the intervention group in discussing difficult topics such as HIV/AIDS and sex (F_1,166_ = 4.83, *p* < 0.05). Social Network Support: The intervention group reported significantly greater social network support post-intervention (F_1,155_ = 7.01, *p* < 0.01).
Brand et al. (2019) [29] Germany	To assess whether supplementing standard informed consent with a comic improves patient comprehension, anxiety, and satisfaction before coronary angiography.	RCT	N = 121 Hospitalized patients undergoing coronary angiography	Health outcomes; patient knowledge of the medical procedure	Baseline (T1) and post-intervention (T2)	Intervention: Standard informed consent + medical graphic narrative (“comic”) (IC comic) Comparison: Control group with standard informed consent only (IC standard)	Patient Comprehension: The IC comic group had significantly better comprehension (mean score = 11.5 ± 1.8 [88.1% correct]) compared to the IC standard group (mean score = 9.1 ± 2.4 [70.2% correct]); difference = −2.3 points (95% CI: −3.08 to −1.56), *p* < 0.001. Periprocedural Anxiety: Mean STAI score increased in the IC standard group by 2.0 points (±5.8) and decreased in the IC comic group by 3.1 points (±6.4); difference = −5.1 points (95% CI: −7.32 to −2.94), *p* < 0.001. Patient Satisfaction: The IC comic group reported higher satisfaction with the IC process (CSQ-8 score = 27.7 ± 3.1) compared to the IC standard group (CSQ-8 score = 25.2 ± 4.2); difference = −2.5 points (95% CI: −3.82 to −1.15), *p* < 0.001.
Cicero et al. (2020) [36] Bolivia	To evaluate an educational comic book-based strategy to improve knowledge, attitudes, and practices (KAP) regarding epilepsy among schoolchildren.	RCT	N = 83 High school students from urban and rural backgrounds in the Chaco region	Behavioral change outcomes (e.g., health self-management behaviors); attitudes and knowledge	Baseline, immediately post-intervention, three-month follow-up	Intervention: Autonomous reading group: students individually read the comic book; character interpretation group: students participated in a group role-playing activity based on the comic book Comparison: None (both groups received the intervention with different methodologies)	Overall KAP Score: Significant improvement in total KAP score (mean ± SD = 9.4 ± 4.3 at baseline vs. 12.5 ± 3.9 post-intervention, *p* < 0.001). Knowledge Subscore: Improved from 2.7 ± 1.7 at baseline to 4.8 ± 1.4 post-intervention (*p* < 0.001). Attitudes Subscore: Improved from 2.6 ± 1.2 at baseline to 3.0 ± 0.9 post-intervention (*p* = 0.004). Practices Subscore: Improved from 3.9 ± 2.3 at baseline to 4.7 ± 2.4 post-intervention (*p* < 0.001). Follow-Up: No significant difference in KAP scores between post-intervention and three-month follow-up.
Farquharson et al. (2023) [74] UK	To assess the efficacy of a brief educational intervention aimed at reducing the patient delay in seeking treatment during acute coronary syndrome (ACS) symptoms.	RCT; 3-armed web-based parallel RCT	N = 145 (93 in analyzed sample) Adults who had experienced ACS within previous 6 months	Behavioral outcome: patient knowledge of ACS symptoms, intended behavior in response to symptoms, actual time taken to seek medical help during subsequent ACS event	Baseline and follow-up post-intervention (timeframe not specified)	Intervention: Usual care + text + visual, usual care + text Comparison: Standard care without educational intervention	Intention to Phone Ambulance: Control: Mean ± SD = 0.32 ± 2.29 (change in score = 0.32, 95% CI [−0.42, 1.06]). Text + Visual: Mean ± SD = 0.68 ± 1.49 (change in score = 0.68, 95% CI [0.18, 1.17], *p* < 0.05). Text Only: Mean ± SD = 0.36 ± 1.91 (change in score = 0.36, 95% CI [−0.29, 1.02]).
Garcia De Avila et al. (2022) [49] Brazil	To analyze the efficacy of verbal guidance given by nurses versus verbal guidance combined with a comic book regarding preoperative anxiety in children and their parents.	Randomized parallel two-group controlled clinical trial	N = 60 Children aged 6–14 years undergoing surgical procedures for the first time and their parents	Health outcomes: preoperative anxiety in children and parents	Pre-intervention and post-intervention (immediately before surgery)	Intervention: Verbal guidance combined with a comic book Comparison: Verbal guidance only	Children’s Anxiety (CAQ Scores): No significant difference between pre- and post-intervention scores in both groups. CAQ scores for the control group were 7.0 (pre-) and 8.9 (post-), while the intervention group had scores of 7.5 (pre-) and 7.0 (post-) (*p* = 0.970). Parents’ Anxiety (HAM-A Scores): Significant decrease in parental anxiety in both groups. The HAM-A score for the control group decreased from 8.0 (pre-) to 4.5 (post-) and for the intervention group from 7.0 (pre-) to 5.0 (post-) (*p* < 0.05). mYPAS Scores (Children’s Anxiety in the OR): No significant difference between groups. Control group median = 6.5; intervention group median = 9.0 (*p* > 0.05).
Gillies et al. (1990) [58] UK	To evaluate the impact of an AIDS education comic on the knowledge, attitudes, and behavioral intentions of 14-year-old school pupils.	RCT	N = 284 14-year-old school students	Behavioral outcome: knowledge, attitudes, and behavioral intentions regarding HIV/AIDS	Baseline, 2 weeks post-intervention	Intervention: AIDS education comic (Streetwize UK) Comparison: No educational intervention during the study period	Knowledge: Significant improvement in knowledge in the experimental group (mean knowledge score: pre-test: 32.5; post-test: 37.8; *p* < 0.001). Attitudes: Improvement in the belief that having one faithful partner can protect against HIV (pre-test: 75%; post-test: 90%; *p* < 0.01). Behavioral Intentions: High levels of intention to use condoms were maintained, but no significant changes in behavioral intentions post-intervention (experimental: 94%; control: 95%).
Gillmore et al. (1997) [37] USA	To assess the impact of a skill-based intervention to encourage condom use among high-risk heterosexually active adolescents.	RCT	N = 396 (received intervention), 306 (1st follow-up), 314 (2nd follow-up) High-risk heterosexually active adolescents aged 14–19 years old	Behavioral outcomes, e.g., condom use, intention to use condoms, self-efficacy in using condoms, and other related behaviors and attitudes	Baseline (pre-intervention) and 12-month follow-up (post-intervention)	Intervention: Skill-based intervention focusing on condom use; comic + video, comic + video + skill Comparison: Standard sex education without focus on condom use skills; comic book only	Condom Use: In the clinic sample, no significant differences in condom use were observed at either follow-up. However, in the detention sample, there was a marginal difference at three months favoring the video condition for condom use with steady partners (F = 2.98; *p* = 0.056), with adjusted means of 2.64 for the comic condition, 3.62 for the video condition, and 3.06 for the group condition. Number of Sexual Partners: No significant differences were observed in the number of sexual partners at either the three-month or six-month follow-ups. Communicating the Desire to Use Condoms: There was a marginal difference at three months in the clinic sample (chi-square = 3.18; *p* = 0.075), where 63.9% of those in the comic condition had suggested using condoms, versus 77.5% in the video condition.
Hill et al. (2016) [50] USA	To evaluate the effect of automated pictograph enhancement on patients’ recall of discharge instructions and satisfaction.	RCT	N = 144 Patients discharged from semi-complicated medical procedures	Health service engagement outcomes; comprehension of discharge instructions, immediate and delayed recall of discharge instructions, patient satisfaction with discharge instructions	Immediately post-discharge (baseline) and one-week post-discharge	Intervention: Pictograph-enhanced discharge instructions Comparison: Standard discharge instructions	Immediate Recall: Patients who received pictograph-enhanced discharge instructions recalled 35% more of their instructions at discharge than those who received standard instructions (pre-recall ratio: standard = 0.04 ± 0.03, pictograph-enhanced = 0.06 ± 0.03, *p* = 0.001). Delayed Recall: No significant difference in recall at one week post-discharge between the two groups (post-recall ratio: standard = 0.04 ± 0.02, pictograph-enhanced = 0.04 ± 0.02, *p* = 0.852). Patient Satisfaction: Higher satisfaction with the understanding of instructions in the pictograph group at one week post-discharge (relative risk = 1.13, 95% CI [0.99, 1.29], *p* = 0.070).
Hutton et al. (2017) [51] USA	To test the efficacy of a specially designed children’s book compared to that of b-ochures regarding safe sleep knowledge and adherence among low socioeconomic-status mothers. RCT: baseline, 1 week, and 2 months postpartum.	RCT	N = 282 Low-socioeconomic-status first-time mothers	Health outcomes (e.g., reduction in SUID); knowledge and adherence	Third trimester (baseline), 1 week postpartum, and 2 months postpartum	Intervention: Children’s book on safe sleep Comparison: Standard brochures on safe sleep	Safe Sleep Knowledge: Children’s Book Group: Safe sleep knowledge increased significantly between prenatal intervention and 1 week (50% increase, *p* < 0.01) and prenatal intervention and 2 months (65% increase, *p* < 0.01) and marginally between 1 week and 2 months (9% increase, *p* = 0.11). Brochure Group: Safe sleep knowledge increased significantly between prenatal intervention and 1 week (36% increase, *p* < 0.01) and prenatal intervention and 2 months (45% increase, *p* < 0.01) and marginally between 1 week and 2 months (7% increase, *p* = 0.11). No significant difference in total knowledge scores between groups at any timepoint. Adherence to Safe Sleep Practices: Bed Sharing: Odds of reported bed sharing (sometimes or often) were significantly higher in brochure group compared to book group (Odds Ratio = 1.81, 95% CI [1.15–2.86], *p* < 0.01). Odds of observed bed sharing were significantly lower in book group (Odds Ratio = 0.44, 95% CI [0.41–0.48], *p* < 0.01). Exclusive Crib Use: Higher increase in exclusive crib use in book group (12% to 29%) compared to brochure group. Usefulness of Materials: Home visitors found book group’s materials facilitated more dialogue, with preference for balance of written content, pictures/graphics, and interactive mode of presentation. Time required for presentation was significantly less for book (mean = 8.81 min) compared to brochure (mean = 10.13 min, *p* < 0.05).
Imamura et al. (2014) [43] Japan	To develop an Internet-based computerized cognitive behavior therapy (iCBT) program in a manga format and examine its effects on improving sub-threshold depression among workers.	RCT	N = 762 Employees of private IT companies	Health outcomes (e.g., depression symptoms, psychological distress, dysfunctional attitudes), behavioral outcomes (e.g., knowledge and self-efficacy regarding cognitive behavioral therapy (CBT) components)	Baseline, 3 months, 6 months	Intervention: Six-week iCBT program delivered via Internet, which included CBT skills like self-monitoring, cognitive restructuring, assertiveness, problem-solving, and relaxation, all presented in manga (Japanese comic) format Comparison: No specific intervention but were provided with emails containing stress management tips	Depression (BDI-II): Significant intervention effect with a small effect size (t = −1.99, *p* < 0.05) (Cohen’s d = −0.16; 95% CI: −0.32 to 0.00) at the 6-month follow-up. Kessler Psychological Distress 6-item Scale (K6): Marginally significant effect (t = −1.72, *p* = 0.09) with a small effect size. Dysfunctional Attitudes (DAs): Significant effect (t = −2.43, *p* = 0.02) with a small effect size. Knowledge and Self-Efficacy Regarding CBT Components: Significant improvements in all knowledge and self-efficacy measures (*p* < 0.05), except for problem-solving efficacy.
Kassai et al. (2016) [31] France	To determine whether a pediatric anesthesia comic information leaflet reduced preoperative anxiety in children undergoing major surgery.	Multicenter open-label randomized controlled parallel group trial	N = 111 Children aged 6–17 undergoing surgery	Health outcomes, e.g., preoperative anxiety levels measured by the State-Trait Anxiety Inventory for Children State subscale (STAIC-S)	Pre-anesthesia visit, day of surgery after pre-anesthesia consultation	Intervention: Comic information leaflet sent to their home in addition to the routine verbal information given by the anesthetist Comparison: Routine verbal information only	Preoperative Anxiety (STAIC-S Scores): Intervention Group: Significant reduction in STAIC-S scores (mean = −2.2, *p* = 0.002). Control Group: Slight increase in STAIC-S scores (mean = 0.90).
Kavin et al. (2010) [52] USA	To develop a diabetes education book, pilot its use, and evaluate its impact on patient care.	Pilot RCT	N = 100 (65 completed) Adult new-onset diabetes patients	Behavioral change outcomes (e.g., diabetes knowledge, emotional distress, self-care behavior); health outcomes: HbA1c levels	Baseline, 4 weeks, 3 months, and 6 months	Intervention: Diabetes education book, diabetes book + brief nurse tutorial on how to use it Comparison: Usual care without any additional educational materials	Diabetes Knowledge: Trends toward improved knowledge in both intervention groups. Book-Only Group: Improvement in PAID scores (−4.33 vs. −4.19 in the control group, *p* = 0.97). Self-Care Behaviors (SDSCA Scores): General Diet Scores: Improvement in the book-only group (1.26 vs. −0.24 in the control group, *p* = 0.034). Exercise Scores: Improvement in the book-only group (0.5 vs. −0.85 in the control group, *p* = 0.010). No significant difference in HbA1c levels between groups.
Ke et al. (2021) [42] Taiwan	To evaluate the efficacy of a decision aid with cartoon images in improving consistency between elderly individuals and their surrogates regarding end-of-life (EOL) care preferences.	RCT; quasi-experimental	N = 110 (55 surrogate–elderly pairs) Elderly patients and their surrogates (family caregivers)	Behavioral change outcomes (e.g., consistency between elderly individuals and surrogates in terms of EOL care preferences and understanding of related terms)	Pre-test and post-test	Intervention: Decision aid with cartoon pictures Comparison: Usual care	Consistency in EOL Preferences: Improvement in consistency between elderly individuals and surrogates in the experimental group, with a significant reduction in the gap between their preferences (mean difference = −8.0, SD = 28.36) compared to the control group (mean difference = −0.3, SD = 27.58). Understanding of Terms: Significant improvement in the understanding of the term “coma” in the experimental group compared to the control group (*p* = 0.036).
Kolberg et al. (2021) [33] USA	To assess the feasibility of using comic-based concussion discharge instructions to improve concussion knowledge and patient satisfaction in pediatric emergency care.	RCT; quasi-experimental	N = 94 (57 patients, 37 guardians) Adolescents aged 11–17 years who presented with a concussion	Behavioral change outcomes (e.g., concussion knowledge, likeability of educational materials, and readability)	Pre-intervention, post-comic, and post-comic + video	Intervention: Comic-only group, comic + video group Comparison: None (both groups received interventions)	Concussion Knowledge: An improvement in concussion knowledge was observed in both groups, with a higher percentage of patients (87.1%) and parents (89.5%) in the comic + video group reporting that they understood more about concussions after the intervention compared to the comic-only group (61.4% of patients and 63.9% of parents). Likeability: The comic handout was liked by 89.5% of patients and 89.2% of parents, while the video was liked by 96.8% of patients and 100% of parents. Readability: The comic handout was found to be “very easy” or “easy” to read by 89.5% of patients and 81.1% of parents.
Kolcak et al. (2023) [75] Turkey	To evaluate the effect of using illustrated materials for communication on the anxiety and comfort levels of patients who received mechanical ventilation after cardiac surgery.	RCT	N = 60 Adult patients who received mechanical ventilation following cardiac surgery	Health outcomes (e.g., pain, anxiety, and comfort levels)	Pre-intervention; 30 min, 60 min, and 1-day post-intervention	Intervention: Illustrated communication materials Comparison: Standard techniques without illustrated materials	Anxiety Levels (Face Anxiety Points Difference): After 30 min of communication, the intervention group showed a significant reduction in anxiety levels, with a mean difference in their face anxiety points of −14.16 compared to −6.11 in the control group (*p* < 0.05). Comfort Levels (Perceived Comfort During Mechanical Ventilation): The mean comfort score in the intervention group was significantly higher (106.10) compared to in the control group (88.53), indicating that patients in the intervention group felt more comfortable during postoperative mechanical ventilation (*p* < 0.05). Satisfaction with the Communication Method: A higher percentage of patients in the intervention group (90%) reported being satisfied with the communication method used compared to the control group (30%), and this difference was statistically significant (*p* < 0.05).
Kovacs et al. (2011) [35] Spain	To evaluate the effect of a simple educational campaign using a comic book on schoolchildren’s knowledge regarding low back pain (LBP) prevention and management.	Cluster RCT	N = 497 8-year-old schoolchildren	Behavioral change outcomes (e.g., knowledge of LBP prevention and management)	Baseline, 15 days post-intervention, 98 days post-intervention	Intervention: Comic book on low back pain prevention Comparison: No comic book (control)	Knowledge Improvement: The percentage of correct answers was above 73% in both groups at baseline. After the intervention, the odds of achieving a score over 80% (considered a “success”) were significantly higher in the intervention group compared to the control group (Odds Ratio = 1.61, 95% CI [1.03–2.52], *p* = 0.038). Sustained Effect: The knowledge improvement was sustained 3 months after the intervention.
Kripalani et al. (2012) [47] USA	To evaluate the effect of graphically enhanced interventions on medication adherence among patients with coronary heart disease (CHD).	RCT; 2 × 2 factorial design	N = 435 Adults with coronary heart disease at an inner-city primary care clinic	Behavioral change outcomes (e.g., medication adherence)	Baseline, 1 year post-intervention	Intervention: Patients received either refill reminder postcards, illustrated daily medication schedules, or both Comparison: Usual care without any additional interventions	Medication Adherence: Both Interventions Group: 36.9% of patients were adherent (cumulative medication gap < 0.20). Illustrated Medication Schedules Group: 34.2% adherence. Refill Reminder Postcards Group: 28.3% adherence. Usual Care Group: 31.2% adherence. Post Hoc Analysis: Patients with more than eight medications or low self-efficacy had significantly greater odds of adherence with illustrated medication schedules (OR = 2.2 and 95% CI [1.21, 4.04] and OR = 2.15 and 95% CI [1.11, 4.16], respectively).
Kulkarni et al. (2022) [32] India	To assess the efficacy of a pediatric anesthesia comic information leaflet in reducing preoperative anxiety in children.	RCT	N = 150 Children aged 6–12 undergoing elective surgery	Health outcomes (e.g., anxiety levels)	Baseline (T0), day prior to surgery (T1), and day of surgery (T2)	Intervention: Comic leaflet + verbal information Comparison: Verbal information only	Preoperative Anxiety (mYPAS Scores): Baseline (T0): No significant difference between groups (T0 scores: 25.8 ± 6.1 in Group T vs. 26.8 ± 8.0 in Group C). Day Prior to Surgery (T1): No significant decrease in anxiety scores (T1 scores: 25.8 ± 5.5 in Group T vs. 26.6 ± 7.8 in Group C, *p* = 0.20). Day of Surgery (T2): No significant difference in anxiety scores (T2 scores: 38.9 ± 14.2 in Group T vs. 41.3 ± 16.4 in Group C, *p* = 0.24). Parental Feedback: 97.3% of parents in the intervention group found the comic leaflet comforting and informative.
Leung et al. (2014) [45] USA	To determine whether a single exposure to a manga comic promoting fruit intake influenced snack selection among minority urban youth.	2-group pilot RCT	N = 57 Black and Hispanic youth aged 8–15 years attending after-school programs	Behavioral change outcomes (e.g., snack selection); knowledge, self-efficacy, and outcome expectations related to fruit intake	Baseline, post-intervention	Intervention: Manga comic promoting fruit intake Comparison: Non-health-related newsletter	Snack Selection: 61% of the comic group chose a healthy snack, compared to 35% in the control group (Odds Ratio = 3.63, 95% CI [1.09–12.1], *p* = 0.04). Self-Efficacy: Significant improvement in self-efficacy in the comic group (mean change = 0.38, SD = 0.92, *p* = 0.04). Knowledge: No significant change in knowledge within the comic group (mean change = 0.24, SD = 0.65, *p* = 0.07). Transportation: The comic group reported significantly higher transportation scores (mean = 3.36, SD = 0.1) compared to the control group (mean = 2.79, SD = 0.1, *p* = 0.006).
Leung et al. (2017) [44] USA	To determine whether exposure to a manga comic with messages promoting fruit consumption influenced psychosocial variables associated with increased fruit intake in middle school youth.	RCT	N = 263 Middle school youth aged approximately 13 years	Behavioral change outcomes (e.g., fruit consumption); self-efficacy, knowledge related to fruit intake, transportation (immersion in media), and enjoyment	Pre-intervention, 4–6 days post-intervention	Intervention: Manga comic promoting fruit consumption Comparison: Newsletter group: participants read a newsletter about fruit Attention control group: participants read a newsletter about ancient Greece	Outcome Expectations: The comic group showed a significant increase in outcome expectations compared to the attention control group (M = 0.44 and SD = 1.64 vs. M = −0.24 and SD = 1.81, t(219) = 2.18, *p* = 0.030). Significant within-group changes in outcome expectations were observed in both the comic group (pre–post M = 8.15 and SD = 1.94 vs. M = 8.59 and SD = 1.86, t(80) = −2.45, *p* = 0.017) and the newsletter group (pre–post M = 7.78 and SD = 1.96 vs. M = 8.36 and SD = 1.86, t(79) = −2.71, *p* = 0.008). Self-Efficacy and Knowledge: No significant differences were observed between the comic group and the other two groups in self-efficacy or knowledge related to fruit consumption (*p* > 0.05). However, within-group changes in knowledge were significant in the comic group (pre–post M = 0.42 and SD = 0.18 vs. M = 0.49 and SD = 0.22, t(80) = −2.60, *p* = 0.011) and the newsletter group (pre–post M = 0.44 and SD = 0.19 vs. M = 0.54 and SD = 0.19, t(163) = −4.42, *p* < 0.001). Transportation: The comic group was significantly more transported by their reading material compared to the newsletter group (M = 3.05 and SD = 0.68 vs. M = 2.78 and SD = 0.51, t(198) = 2.49, *p* = 0.014) and the attention control group (M = 3.05 and SD = 0.68 vs. M = 2.55 and SD = 0.57, t(198) = 5.05, *p* < 0.001). Enjoyment: Participants in the comic group reported greater enjoyment with their reading material than the newsletter group (M = 7.07 and SD = 2.81 vs. M = 5.98 and SD = 2.57, t(220) = 2.38, *p* = 0.018) and the attention control group (M = 7.07 and SD = 2.81 vs. M = 6.17 and SD = 2.43, t(220) = 1.96, *p* = 0.030).
Lusiana et al. (2023) [30] Indonesia	To evaluate the efficacy of a health education program using a “SIMON” comic with a Papua-emic approach to improving the food intake of breakfast and vegetables among elementary school students.	RCT; quasi-experimental	N = 60 Elementary school students in grades 3 and 4	Behavioral change outcomes (e.g., breakfast and vegetable consumption); knowledge	Baseline and 1 month post-intervention	Intervention: Comic (SIMON) on breakfast and vegetable consumption Comparison: Leaflet with the same nutritional message	Knowledge: In the comic group, 19 students (mean rank = 13.74) showed an increase in knowledge post-intervention, while 5 students (mean rank = 7.80) showed a decrease, and 6 students remained the same. The difference was statistically significant (*p* = 0.001). In the leaflet group, 15 students (mean rank = 12.33) showed an increase in knowledge, while 8 students (mean rank = 11.38) showed a decrease, and 7 students remained the same. This difference was not statistically significant (*p* = 0.15). Attitudes: In the comic group, 14 students (mean rank = 10.64) showed an improvement in attitudes, 6 students (mean rank = 10.17) showed a decrease, and 10 students remained the same. The change was not statistically significant (*p* = 0.096). In the leaflet group, 12 students (mean rank = 11.20) showed an improvement in attitudes, 10 students (mean rank = 11.85) showed a decrease, and 8 students remained the same. The difference was not statistically significant (*p* = 0.79). Behavior: In the comic group, 11 students (mean rank = 9.14) showed an improvement in behavior, 6 students (mean rank = 8.75) showed a decrease, and 13 students remained the same. The change was not statistically significant (*p* = 0.242). In the leaflet group, 10 students (mean rank = 8.10) showed an improvement in behavior, 4 students (mean rank = 6.00) showed a decrease, and 16 students remained the same. The difference was marginally significant (*p* = 0.06)
Monroe et al. (2018) [53] USA	To evaluate the efficacy of a pictorial aid intervention regarding medication adherence among patients living with HIV and comorbid conditions, specifically hypertension (HTN) and diabetes mellitus (DM).	RCT	N = 46 Patients with diagnosed HIV and either diabetes or hypertension	Behavioral change outcomes (e.g., medication adherence)	Baseline and follow-up	Intervention: Pictorial medication aid Comparison: Standard clinic visit discharge list	Medication Understanding: The average score for medication understanding was 0.78, with 63.0% of participants scoring 0.80 or higher. Medication Adherence (MPR): ART (Antiretroviral Therapy): Baseline median MPR = 92% (IQR 71–100%). Follow-up median MPR = 89% (IQR 72–100%). Mean change in the MPR for ART in the intervention group was 0.02 (SD = 0.25), and in the control group, it was 0.02 (SD = 0.33), with no significant difference between groups (*p* = 0.96). Diabetes or Hypertension Medications: Baseline median MPR = 79% (IQR 63–96%). Follow-up median MPR = 85% (IQR 65–98%). Mean change in the MPR for diabetes or hypertension medications in the intervention group was −0.02 (SD = 0.31), and in the control group, it was 0.08 (SD = 0.45), with no significant difference between groups (*p* = 0.32). Participant Satisfaction: High satisfaction with the intervention, with a mean score of 24.8 (SD = 4.0) on a 28-point scale. Perceived Helpfulness: Participants rated the aid as most helpful in remembering what medications to take and the names of their medications (mean score = 2.9 and SD = 0.3 on a 3-point scale). Ease of Understanding: High ratings for easy-to-understand instructions (mean = 3.7 and SD = 0.7 on a 4-point scale) and clear pictures depicting the medication’s purpose (mean = 3.7 and SD = 0.5 on a 4-point scale).
Montgomery et al. (2004) [48] UK	To investigate the efficacy of a brief media-based behavioral treatment for sleep problems in young learning-disabled children.	RCT	N = 66 Children aged 2–8 with severe learning disabilities and sleep problems	Behavioral change outcomes (e.g., sleep habits); health outcomes	Baseline, post-intervention, and six-month follow-up	Intervention: Booklet group: received a 14-page illustrated booklet on sleep management techniques Face-to-face group: received conventional face-to-face behavioral treatment Comparison: No immediate intervention	Composite Sleep Disturbance Scores: Significant improvements were found in both intervention groups compared to the control group (H = 34.174, df = 2, *p* < 0.001). Post-treatment scores: face-to-face: mean = 2.4, SD = 1.93; booklet: mean = 2.55, SD = 2.76; control: mean = 5.75, SD = 1.54. Six-Month Follow-Up: Improvements in sleep disturbance were maintained at six months (face-to-face: mean = 1.89, SD = 2.02; booklet: mean = 2.08, SD = 2.89).
Nasir et al. (2018) [76] Pakistan	To compare the effects of play distraction versus pharmacological treatment on anxiety levels in children undergoing day surgery.	RCT	N = 240 Children undergoing day surgery	Health outcomes (e.g., anxiety levels)	Baseline and post-intervention	Intervention: Play distraction using a comic book Comparison: Pharmacological premedication (Midazolam)	Modified Yale Preoperative Anxiety Scale (mYPAS) Scores: The mean total mYPAS score for the intervention group was 10.74 ± 1.18, and for the control group, it was 10.55 ± 0.91. The mean difference was 0.186, which was statistically insignificant (*p* = 0.172). STAIC Scores: The mean total STAIC score for the intervention group was 20.88 ± 2.24, and for the control group, it was 20.85 ± 2.07. The mean difference was 0.037, which was statistically insignificant (*p* = 0.894).
Negarandeh et al. (2013) [55] Iran	To compare the efficacy of the teach-back method and pictorial image education strategies regarding diabetes-specific knowledge, adherence to medication regimens, and adherence to dietary regimens among patients with low health literacy.	RCT	N = 127 Patients with type 2 diabetes and low health literacy	Behavioral change outcomes (e.g., medication adherence, dietary adherence); knowledge	Baseline and follow-up (exact timepoint not specified)	Intervention: Pictorial image group: received education using pictorial images Teach-back group: received education using the teach-back method Comparison: Control group with standard care without additional educational interventions	Health Literacy: The Test of Functional Health Literacy in Adults (TOFHLA) scores indicated low health literacy among participants across all groups: the pictorial image group (M = 34.84, SD = 15.70), teach-back group (M = 34.71, SD = 14.60), and control group (M = 33.58, SD = 16.46). Diabetes-Specific Knowledge: An ANOVA showed significant differences in knowledge improvement between the three groups at follow-up (*p* < 0.05). A Tukey HSD test confirmed significant differences between both intervention groups and the control group, with no significant difference between the two intervention groups. Adherence to Medication and Dietary Regimen: Significant improvements were observed in adherence scores in all groups at follow-up. However, the magnitude of improvement was significantly greater in the intervention groups compared to in the control group (*p* < 0.05). Overall Impact: The differences between the baseline and follow-up measurements demonstrated that the intervention groups had a more pronounced improvement in diabetes-specific knowledge and adherence to both medication and dietary regimens compared to the control group (*p* < 0.05).
Nestadt et al. (2019) [41] Thailand	To evaluate the short- and long-term impact of a pilot RCT of the CHAMP+ Thailand (Collaborative HIV/AIDS and Adolescent Mental Health Program) on a range of behavioral, health, psychosocial, and family factors.	RCT	N = 88 pairs Children (9–14 years old) with perinatal HIV and their caregivers	Behavioral change outcomes (e.g., youth–caregiver communication, stigma reduction); health outcomes (e.g., HIV knowledge)	Baseline, 6 months, 9 months	Intervention: CHAMP+ Thailand Comparison: Standard of care (SOC)	Youth mental health: Significant improvement in SDQ total difficulty scores in CHAMP+ group (mean score change: −0.82 [6 months] to −3.98 [9 months]; *p* < 0.05). ART adherence: 90% viral suppression at baseline, maintained post-intervention. HIV knowledge: Improved in both youth and caregivers, significantly better in CHAMP+ group (caregiver knowledge OR = 0.4, *p* < 0.05). HIV stigma: Caregiver-reported internalized stigma improved significantly in CHAMP+ group (mean score change: −1.98, *p* < 0.05). Social support: Caregiver-reported HIV-related social support improved significantly (mean score change: +0.4, *p* < 0.05). Youth–caregiver communication: Improved significantly in CHAMP+ group, sustained at 9 months.
Niu et al. (2022) [77] USA	To evaluate the efficacy of digital educational strategies for teaching skin self-examination (SSE) to melanoma survivors.	RCT; 3 × 2 between-subjects factorial design using an online experimental approach	N = 321 Adults at risk for skin cancer	Behavioral change outcomes (e.g., SSE intention and accuracy); knowledge	Post-intervention	Intervention: Various combinations of interactivity (high vs. low) and character imagery (cartoon, real person, customization) Comparison: Low interactivity and cartoon imagery	Accuracy: High-interactivity and customization group (M = 4.37, SE = 0.22) showed better accuracy in identifying abnormal skin lesions compared to low-interactivity and cartoon group (M = 3.71, SE = 0.22) (*p* < 0.05). SSE Intention: Higher intention in high-interactivity and customization (M = 3.28, SE = 0.16) and low-interactivity and real human character groups (M = 3.27, SE = 0.16) compared to low-interactivity and cartoon group (M = 2.80, SE = 0.16) (*p* < 0.05)
Seeliger et al. (2022) [56] Germany	To evaluate the impact of a graphic narrative supplement on patient satisfaction and anxiety during the provision of informed consent for bronchoscopy in lung transplant patients.	RCT	N = 59 Adults and pediatric patients transitioning to adult care undergoing bronchoscopy after lung transplantation	Health outcomes (e.g., anxiety measured by STAI, adverse experiences during bronchoscopy); behavioral outcome: patient satisfaction with informed consent	Baseline, post-consent, post-procedure	Intervention: Graphic narrative + informed consent Comparison: Standard informed consent	Patient Satisfaction: Overall satisfaction with the informed consent was significantly higher in the intervention group (median = 9.5; 25Q–75Q: 8.6–9.8) compared to the control group (median = 8.6; 25Q–75Q: 8.1–9.2), *p* = 0.028. Recommendation of Materials: Patients in the intervention group were more likely to recommend the information material to others (median score = 9.5 vs. 8.0, *p* = 0.038). Engagement with Materials: The intervention group had a higher median score for reading through the complete information material compared to the control group (median = 9.6 vs. 6.3, *p* = 0.009). Anxiety (STAI): There was no significant difference in the change in STAI scores between the intervention and control groups (median change = 0.5 vs. 1, *p* = 0.972). Patient Experience During Bronchoscopy: No significant differences were reported in the patient-reported anxiety, dyspnea, coughing, gagging, pain, or discomfort between the groups. Bronchoscopy Assistant’s Assessment: The bronchoscopy assistant judged the overall patient discomfort during bronchoscopy to be lower in the intervention group compared to in controls (median score = 3 vs. 4, *p* = 0.040). Physician’s Assessment: The physician rated the relaxation level of patients higher in the intervention group (median score = 7 vs. 6, *p* = 0.037).
Shin et al. (2022) [38] USA	To describe the impact of a comic book on adolescents’ knowledge, beliefs, and intentions regarding HPV vaccination.	RCT; quasi-experimental	N = 136 East African immigrant adolescents aged 14–17 years old	Behavioral outcomes (e.g., HPV vaccine-related knowledge, beliefs, intentions)	Pre- and post-intervention	Intervention: Comic book on HPV/HPV vaccine Comparison: Standard education, no comic book	HPV knowledge increased significantly in both genders post-intervention (females: 47.6 (6.3) pre-intervention, 82.5 (3.6) post-intervention, RR = 1.73, 95% CI: 1.33–2.24; males: 40.6 (5.4) pre-intervention, 83.6 (4.6) post-intervention, RR = 2.07, 95% CI: 1.56–2.75).
Tekle-Haimanot et al. (2016) [34] Ethiopia	To assess the impact of an educational comic book on epilepsy-related knowledge, attitudes, and practices among schoolchildren in Ethiopia.	RCT	N = 226 High school students from urban and rural areas in Ethiopia	Behavioral change outcomes (e.g., knowledge, attitudes, and practices regarding epilepsy)	Pre- and post-intervention	Intervention: Comic book on epilepsy No comparison group	Significant improvements in knowledge after reading the comic book: urban school—knowledge of epilepsy causes increased from 13.1% to 82.7% (*p* < 0.001); rural school—increased from 53% to 87% (*p* < 0.001); awareness that epilepsy is not contagious increased to 87.1% in urban and 70.9% in rural schools (*p* < 0.001).
Tjiam et al. (2013) [57] The Netherlands	To compare the effects of three tools (educational cartoon story, reward calendar, and information leaflet) on compliance with occlusion therapy in children with amblyopia.	RCT; compliance measured over 1 week	N = 88 3- to 6-year-old children in low-socioeconomic-status (SES) areas starting occlusion therapy for amblyopia	Behavioral change outcomes: compliance with occlusion therapy (primary); actual occlusion hours per day (secondary)	Baseline, post-intervention (1 week)	Intervention: Educational cartoon story; reward calendar; information leaflet for parents Comparison: Coloring pictures	The educational cartoon story group showed the highest compliance (89%, *p* = 0.002) compared to the control group (55%). The reward calendar and information leaflet groups had compliance rates of 67% (*p* = 0.301) and 73% (*p* = 0.119), respectively. The cartoon group also had the highest actual occlusion hours per day.
Zhou et al. (2023) [61] USA	To test the efficacy of narrative messages for critical health communication in influencing health-related knowledge, attitudes, and behaviors.	RCT	N = 129 Mexican American women aged 18–29	Behavioral change outcomes: intentions to reduce sugary beverage consumption, media literacy, public health literacy, empowerment	Baseline, post-intervention (1 week)	Intervention: Narrative message in video format; narrative message in comic book format No comparison group (within-group comparision)	Both narrative messages (video and comic) significantly increased intentions to reduce sugary beverage consumption (video: *p* < 0.01, d = 0.43; comic: *p* = 0.03, d = 0.28). Both formats also improved media literacy (video: *p* = 0.01, d = 0.34; comic: *p* = 0.05, d = 0.25), public health literacy (video: *p* = 0.05, d = 0.24; comic: *p* = 0.01, d = 0.32), and empowerment to engage in community movements (video: *p* = 0.003, d = 0.38; comic: *p* = 0.034, d = 0.27).
